# Membrane-associated estrogen receptor α prevents the amyloid β-induced suppression of GIRK channel activity in hippocampal neurons from female mice

**DOI:** 10.1186/s13293-025-00776-7

**Published:** 2025-11-06

**Authors:** Haichang Luo, Ezequiel Marron Fernandez de Velasco, Jaeyoon Kim, Praseuth Yang, Paul Mermelstein, Joseph V. Bonventre, Paul S. Cooke, Kevin Wickman

**Affiliations:** 1 Department of Pharmacology, University of Minnesota, 6-120 Jackson Hall, 321 Church Street SE, Minneapolis, 55455 MN USA; 2 Department of Neuroscience, University of Minnesota, Minneapolis, 55455 MN USA; 3 Division of Renal Medicine, Brigham and Women’s Hospital, Boston, 02115 MA USA; 4 Harvard Stem Cell Institute, Harvard Medical School, Boston, 02115 MA USA; 5 Department of Physiological Sciences, University of Florida, Gainesville, 32610 FL USA

**Keywords:** Alzheimer’s disease, Estrogen, Kir3, mGluR5, cPLA2α_2_α, Sex difference

## Abstract

**Background:**

Amyloid β oligomers (oAβ) are a key pathogenic driver in Alzheimer’s Disease (AD). Neuronal G protein-gated inwardly rectifying K^+^ (GIRK/Kir3) channels are important regulators of neuronal excitability and prominent somatodendritic effectors for inhibitory G protein-coupled receptors, including the γ-aminobutyric acid type B receptor (GABA_B_R). We previously reported a male-specific suppression of GIRK channel activity in hippocampal (HPC) neurons evoked by oAβ in in vitro, ex vivo, and in vivo mouse models of AD, and showed that this adaptation correlated with synaptic and cognitive impairment. Using pharmacological approaches, we showed that this adaptation is mediated by co-activation of cellular prion protein (PrP^C^) and metabotropic glutamate receptor 5 (mGluR5) and requires activation of cytosolic phospholipase A2 α (cPLA_2_α). However, the mechanisms underlying the sex specificity was unknown. Given the clinical context that females exhibit a 2-fold higher incidence of AD than males, and the loss of neuroprotective estrogen by menopause contributes to the sex differences in AD, we postulated that estrogen-associated resilience underlies this sex dimorphism of oAβ action.

**Methods:**

To examine the strength of GIRK-dependent signaling in HPC neurons, we performed electrophysiology in primary HPC cultures from neonatal male and female mice and then measured whole-cell currents evoked by the direct-acting GIRK channel agonist ML297 and the GABA_B_R-selective agonist baclofen. We used an array of genetic and pharmacological approaches to investigate the molecular mechanism(s) underlying the vulnerability and resilience of GIRK channel activity to oAβ in male and female HPC neurons, respectively.

**Results:**

We found that resilience to the oAβ-induced and PrP^C^/mGluR5-dependent suppression of GIRK channel activity in female HPC neurons is conferred by membrane-associated estrogen receptor α (mERα) and caveolin 1 (Cav1). When this resilience factor is blocked or absent, oAβ suppresses GIRK channel activity in female HPC neurons via the same PrP^C^-mGluR5-cPLA_2_α signaling pathway identified previously in male neurons.

**Conclusion:**

As estrogen levels decline with aging and menopause, the protective influence of mERα/Cav1 may diminish, unmasking the oAβ-induced suppression of GIRK channel activity and exacerbating disease progression in females.

**Plain english summary:**

While amyloid β plaques (Aβ) are notable hallmarks of Alzheimer’s Disease (AD), cognitive impairment in the early stages of the disease tracks more closely with the level of soluble Aβ oligomers (oAβ) in the brain. oAβ promotes cognitive deficits by disrupting the balance of excitatory and inhibitory influences on neurons in brain regions important for learning and memory such as the hippocampus, but the underlying molecular targets of oAβ and its pathogenic mechanisms are not fully understood. We recently demonstrated that oAβ weakens the activity of a prominent inhibitory influence on neuronal excitability (the GIRK channel) in the hippocampus of male but not female mice. This sexually dimorphic effect of oAβ was interesting and unexpected given that women are twice as likely to develop AD than men, and because disease progression is more aggressive in women. In this study, we investigated the mechanisms underlying the resilience of GIRK channels in female hippocampal neurons to oAβ. We found that resilience is conferred by estrogen and one of its receptors. When the influence of this receptor is diminished using pharmacological or genetic interventions, oAβ weakens GIRK channel activity in female and male neurons to a similar degree, and via the same mechanism. We speculate that with the onset of menopause, the protective influence of estrogen on GIRK channel activity in the hippocampus begins to wane. This, combined with other female-specific effects of oAβ on neuronal activity, contributes to the increased incidence and severity of AD in females.

**Supplementary Information:**

The online version contains supplementary material available at 10.1186/s13293-025-00776-7.

## Highlights

- In hippocampal (HPC) neurons from female mice, local estrogen production protects GIRK channel activity from suppression by oligomeric amyloid β (oAβ).
- Inhibition of estrogen receptor α (ERα), but not estrogen receptor β (ERβ) or G protein-coupled estrogen receptor (GPER), unmasked the oAβ-induced suppression of GIRK channel activity in female HPC neurons.
- Without ERα protection, oAβ suppressed GIRK channel activity in female HPC neurons through the same mechanism identified previously in male HPC neurons involving cellular prion protein (PrP^C^), metabotropic glutamate receptor 5 (mGluR5), and cytosolic phospholipase A_2_α (cPLA_2_α).
- mGluR5-associated regulation of GIRK channel plasticity is blocked in female HPC neurons independent of oAβ.
- Resilience of GIRK channel activity in female HPC neurons to mGluR5 agonist and oAβ requires membrane-associated ERα (mERα) and caveolin-1 (Cav1).

**Supplementary Information:**

The online version contains supplementary material available at 10.1186/s13293-025-00776-7.

## Background

Alzheimer’s disease (AD) is a neurodegenerative disorder and the most common form of dementia [1]. Pathological hallmarks of AD include amyloid plaques, neurofibrillary tangles, neuroinflammation, and gliosis [1]. Disruption of the balance between neuronal excitation and inhibition (E/I) is strongly associated with synaptic loss, a key driver of cognitive decline and neurodegeneration in AD [2]. The hippocampus (HPC), a critical region for cognitive function, is notably vulnerable, exhibiting early hyperactivity followed by progressive atrophy in AD [3, 4].

The main component of plaques is amyloid beta (Aβ), which derives from the cleavage of amyloid precursor protein (APP) by β- and γ-secretases [5]. Among the various Aβ species generated through amyloid precursor protein (APP) cleavage, soluble oligomeric Aβ (oAβ) is regarded as the most toxic and pathogenic [6]. oAβ is thought to trigger the network excitability and synaptic dysfunction that underlie cognitive impairment in early stages of AD [5, 7–13]. Indeed, the onset and severity of cognitive impairment in AD, and the epileptiform activity preceding cognitive decline [14, 15], correlate more strongly with oAβ level than plaques [16]. While the impact of oAβ on excitatory glutamatergic transmission has been extensively characterized [17], its effects on inhibitory signaling are less well-understood [18].

We recently reported that oAβ evokes a male-specific suppression of G protein-gated inwardly rectifying K^+^ (GIRK/Kir3) channel activity in excitatory/glutamatergic HPC neurons in in vitro, ex vivo, and in vivo mouse models [19]. GIRK channels are key regulators of neuronal excitability and prominent somatodendritic effectors for inhibitory G protein-coupled receptors (GPCRs) including the γ-aminobutyric acid type B receptor (GABA_B_R), 5HT_1A_ receptor, and adenosine A_1_ receptor [20]. Either gain- or loss-of-function manipulations of GIRK channel activity in rodents can impair cognitive function [21], and dysregulation of GIRK channel activity has been implicated in many neurological disorders and diseases, including AD [20, 22–24]. For example, aberrant expression and/or subcellular trafficking of GIRK channels in the HPC has been reported in APP/PS1 and other transgenic AD models [25–27], and GIRK-dependent signaling is diminished in CA1 pyramidal neurons of the dorsal HPC in male APP/PS1 mice [19].

There is a 2-fold higher incidence of AD in females than males [28], and disease progression is more aggressive in females post-menopause [29–32]. Although longer lifespan in females may contribute to this disparity, biological sex also influences risk factors and disease mechanisms [33]. Given this clinical context, the resilience of GIRK channel activity in female HPC neurons seen in acute amyloidopathy and transgenic AD models is surprising. Estrogen and its receptors are intriguing potential resilience factors [34]. Estrogen levels in the female brain tend to mirror circulating estrogen levels and are significantly reduced in female brains after menopause [35]. As such, the abrupt waning of this protective influence during perimenopause could contribute to the increased onset and severity of AD in women. Consistent with this premise, estrogen replacement is used as a therapeutic strategy for post-menopausal females with AD [31], and estrogens have neuroprotective effects in rodent AD models [36]. Estrogen replacement improves HPC-dependent cognitive function in rodents [37–42].

Here, we used genetic and pharmacological approaches in the HPC culture model to investigate the molecular mechanism(s) underlying the vulnerability and resilience of GIRK channel activity to oAβ in male and female HPC neurons, respectively. We found that resilience in female HPC neurons is conferred by membrane-associated estrogen receptor α (mERα) and caveolin-1. In the absence of this resilience factor, oAβ suppresses GIRK channel activity in female HPC neurons via the same mechanism observed in male neurons.

## Methods

### Animals

C57BL/6J parents (RRID: IMSR_JAX:000664) were purchased from The Jackson Laboratory and the mice were bred in house. The generation and characterization of nuclear-only ERα (NOERα) mice [43] and constitutive cytosolic phospholipase A_2_α knockout (cPLA_2_α^–/–^) mice [44] were described previously. Caveolin 1 knockout (Cav1^–/–^) mice [45] were obtained from The Jackson Laboratory (B6.Cg-Cav1^tm1Mls^/J, RRID: IMSR_JAX:007083). Male (2–4 per cage) and female (2–5 per cage) mice were group-housed on a 14/10 h light/dark cycle (lights on from 0600 to 2000 h) and given free access to water and food. All animal experiments were approved by the University of Minnesota Institutional Animal Care and Use Committee.

## Reagents

Oligomers of Aβ_1−42_ were prepared and validated as described [19]. In brief, we equilibrated synthetic Aβ_1−42_ (Bachem; Vista, CA, United States) at room temperature for 20 min and resuspended with 1,1,1,3,3,3-hexafluoro-2-propanol (Sigma-Aldrich; St. Louis, MO, United States) to make a 1 mM stock. Solvent in the aliquots was evaporated in a biosafety cabinet for 2–2.5 h, and the resulting peptide films were stored at −80 °C. At the night before use, peptide films were resuspended to 1 mM in dimethyl sulfoxide by 10 min bath sonication, diluted to 100 µM in cold phosphate-buffered saline (PBS) and vortexed for 30 s, then incubated at 4 °C overnight. Oligomer solutions were diluted in culture media to their final concentrations. All the stored films were used within 4 months. (R, S)-CHPG sodium salt, and MTEP were purchased from Abcam (Cambridge, MA, United States); 6D11 (anti-CD230) was purchased from BioLegend (San Diego, CA, United States); (S)−3,5-DHPG, tamoxifen citrate, G-15, and aminoglutethimide were purchased from Cayman Chemical (Ann Arbor, MI, United States); LY367385 hydrochloride and PHTPP were purchased from MedChemExpress (Monmouth Junction, NJ, United States); 2-APB and baclofen were purchased from Sigma-Aldrich; MPP dihydrochloride, 17β-estradiol (E2), and tetrodotoxin were purchased from Tocris (Minneapolis, MN, United States); ML297 was a generous gift from Dr. C. David Weaver at Vanderbilt University. All drugs were stored, handled, and resuspended according to provider specifications. High-titer (>1 × 10^12^ gene copies/mL) AAVPHP.eB-CaMKIIα-jGCaMP8s (plasmid was a gift from Loren Looger; RRID: Addgene_176752) was generated and purified in-house by the University of Minnesota Viral Vector and Cloning Core (Minneapolis, MN).

## Cell culture

Primary HPC neuron cultures were prepared from neonatal (P0-2) C57BL/6J pups, as described [46]. Pup sex was determined by the presence of a pigment spot on the scrotum [47]. Hippocampi were extracted and placed into ice-cold modified Hank’s Balanced Salt Solution (HBSS, Ca^2+^ and Mg^2+^ free, with 1 mM HEPES) containing 20% FBS, rinsed twice with FBS-free HBSS, digested for 20 min at 37 °C with occasional inversion using papain (2.5% v/v) and DNase I (0.1% v/v) in digestion solution (137 mM NaCl, 0.5 mM KCl, 0.7 mM Na_2_PO_4_, 2.5 mM HEPES, pH 7.2). Tissue was then mechanically dissociated by pipetting in Neurobasal A-based plating medium (with 1x B27, 1x Glutamax, 1x antibiotic/antimycotic, and 0.05% DNase I). Cells were pelleted by centrifugation (2500 rpm/587 rcf for 10 min at room temperature). Cells were diluted with plating media accordingly and plated onto poly-L-Lysine (0.0005%) pre-coated 8-mm glass coverslips in 48-well plates for single-cell electrophysiological analysis, or black 96-well plates with transparent bottoms (Thermo Fisher; Waltham, MA, United States) for fluorescence assay. Before experimentation, cultures were maintained in a humidified 5% CO_2_ incubator at 37 °C for 12–14 d, and half of the medium was replaced with fresh growth medium (Neurobasal A with 1x B27, 1x Glutamax, and 1x antibiotic/antimycotic) every 3 d.

## Electrophysiology

Whole-cell patch-clamp recordings in cultured HPC neurons were performed as described [48]. In brief, coverslips with neurons were transferred to a chamber containing a low K^+^ bath solution (130 mM NaCl, 5.4 mM KCl, 1 mM CaCl_2_, 1 mM MgCl_2_, 5.5 mM D-Glucose, 5 mM HEPES, pH 7.4). To target excitatory neurons, neurons with pyramidal morphology and capacitance values between 100 and 200 pF were targeted for analysis. Fire-polished borosilicate patch pipettes (4–6 MW) were filled with K-gluconate internal solution (140 mM K-gluconate, 2 mM MgCl_2_, 1.1 mM EGTA, 5 mM HEPES, 2 mM Na_2_-ATP, 0.3 mM Na-GTP, and 5 mM phosphocreatine, pH 7.2). After achieving whole-cell access, neurons were held at −70 mV; liquid-junction potential was not corrected. A stable recording was first acquired in the low K^+^ bath solution. The drug-evoked whole-cell currents were measured in a high-K^+^ bath solution (120 mM NaCl, 25 mM KCl, 1 mM CaCl_2_, 1 mM MgCl_2_, 5.5 mM D-glucose, 5 mM HEPES, pH 7.4). ML297 and baclofen were diluted in the high-K^+^ bath solution and perfused directly onto the neuron using the ValveLink 8.2 rapid perfusion system (AutoMate Scientific). Whole-cell currents were acquired with an Axopatch-200B amplifier and pCLAMP v.8.2 software (Molecular Devices, LLC). All currents were low-pass filtered at 2 kHz, digitized at 10 kHz with a Digidata 1322 A (Molecular Devices, LLC), and stored on a computer hard disk for subsequent analysis. Only experiments in which the access resistance (R_A_) was stable (change pre- and post-perfusion < 20%) and low (< 15 MW) were included in the analysis. Current density (pA/pF) was calculated as the ratio of current amplitude to cell capacitance. In each set of electrophysiological experiments, the analyzed neurons were acquired from cultures from at least 3 different litters.

## Ca^2+^ imaging

Primary HPC neuronal cultures were prepared from neonatal (P0-2) male and female C57BL/6J pups in a black 96-well plate, plated at a density of 20,000 cells/well. On DIV3, cultured HPC neurons were infected with AAVPHP.eB-CaMKIIα-jGCaMP8s and were maintained in a humidified 5% CO_2_ incubator at 37 °C for 14 d. Half of the medium was replaced with fresh growth medium (Neurobasal A with 1x B27, 1x Glutamax, and 1x antibiotic/antimycotic) every 3d. On DIV17, culture medium was aspirated, and each well was filled with 85 µL of HBSS (containing Ca^2+^ and Mg^2+^) with tetrodotoxin (0.5 µM). The plate was incubated for 10 min at 37 °C and subsequently transferred into the plate reader (CLARIO Star Plus; BMG LabTech; Ortenberg, Germany) preset to 37 °C. Fluorescent intensity was measured from the bottom of each well, with readings taken every 5 s per interval. The baseline fluorescence was measured for 30 s (6 intervals), and 15 µL of vehicle or CHPG (1 mM) was injected into the well. Immediately after injection, the plate reader was programmed to shake the plate in a double orbital mode at 300 rpm. The fluorescence was further measured for 60 s after the drug injection.

### Data analysis

Statistical analyses for all electrophysiological experiments were conducted with Prism v10.4.2 (GraphPad Software; San Diego, CA, United States) using unpaired t-test or ANOVA (one-way or two-way) and post hoc comparisons including Šídák’s, Dunnett’s, Tukey’s multiple comparisons, as appropriate. Statistical analysis for calcium assays was performed in SPSS Statistics v30.0.0.0 (IBM Corporation; Armonk, NY, United States) using nested two-way ANOVA and post hoc Šídák’s multiple comparison test. The distribution of data in all reported datasets was tested before final statistical analysis to ensure the use of appropriate tests.

## Results

### Protective influence of E2/ERα on GIRK channel activity in female HPC neurons

As noted above, estrogens exhibit neuroprotective effects in rodent AD models [36]. Aromatase expression in the HPC allows for local estrogen production [49, 50]. Pharmacological inhibition of aromatase dose-dependently reduced levels of 17-β-estradiol (E2), the major product of estrogen biosynthesis and most potent estrogen [49], in both neonatal rat HPC dispersion and organotypic slice cultures [51]. Moreover, estrogen receptors are expressed in neonatal rodent HPC cultures and are responsive to E2 [52]. Thus, local estrogen production and estrogen receptors could explain the resilience to oAβ-induced suppression of GIRK channel activity in female HPC neurons.

**Fig. 1:**
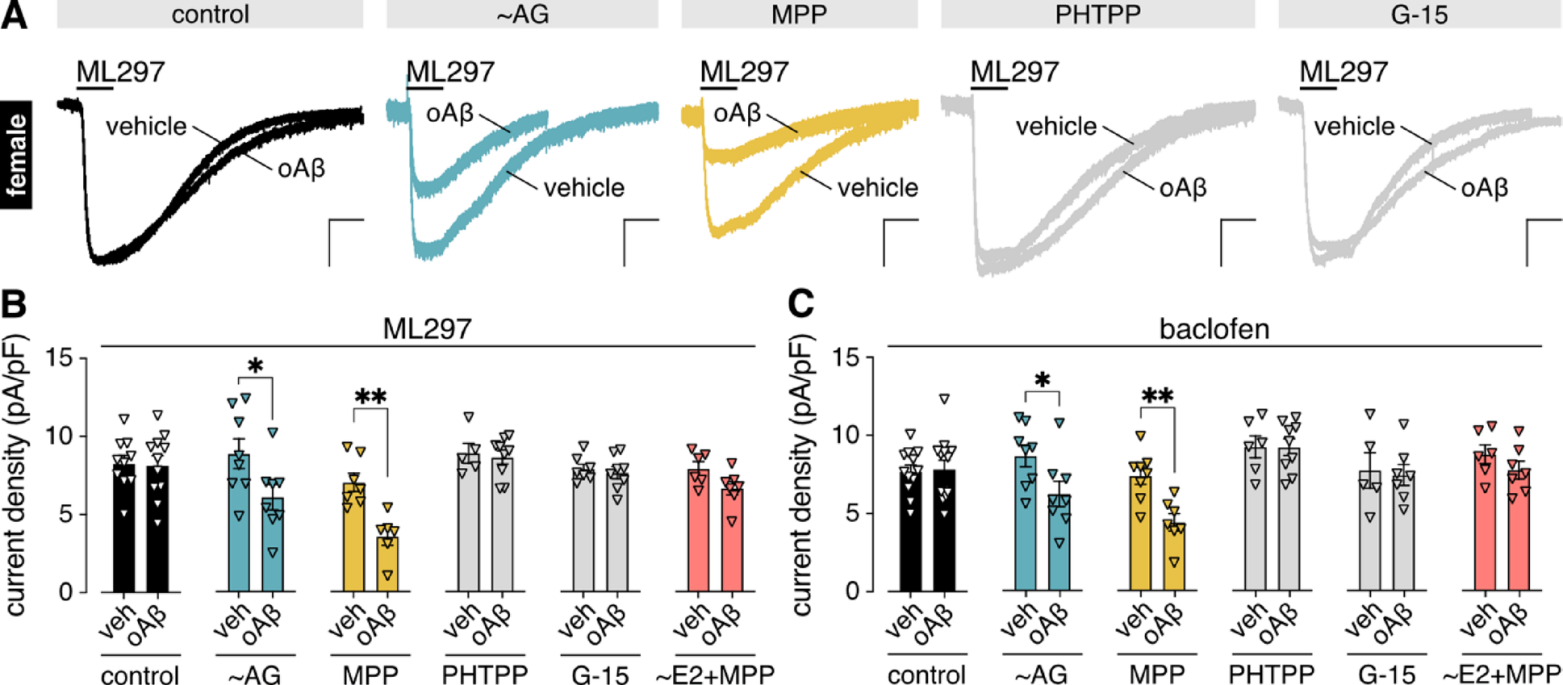
E2/ERα prevents the oAβ-induced suppression of GIRK channel activity in female HPC neurons. **A**. representative currents evoked by ML297 (10 µM) in female HPC neurons treated with vehicle or oAβ (0.5 µM, 3–6 h) without (control) or with the following interventions: (i) supplementation of culture media with the aromatase inhibitor aminoglutethimide (~ AG, 50 µM) from DIV3 onward; (ii) 30-min pretreatment with the ERα antagonist MPP dihydrochloride (MPP, 1 µM), ERβ antagonist PHTPP (1 µM), or GPER antagonist G-15 (1 µM); scale: 500 pA/10 s. **B**. summary of ML297 (10 µM) current densities in female HPC neurons treated with vehicle (veh) or oAβ (0.5 µM, 3–6 h) under control conditions (*n* = 10–11/group); with AG (50 µM) supplementation from DIV3 onward; after a 30-min pretreatment with MPP (1 µM; *n* = 6–7/group), PHTPP (1 µM; *n* = 5–9/group), or G-15 (1 µM; *n* = 7–8/group); or with MPP pretreatment (1 µM, 30 min) of cultures supplemented with 17β-estradiol (~ E2, 1 µM) from DIV3 onward (*n* = 6–7/group). **C**. summary of baclofen (10 µM) current densities in female HPC neurons treated with vehicle (veh) or oAβ (0.5 µM, 3–6 h) under control conditions (*n* = 12–13/group); with AG (50 µM) supplementation from DIV3 onward (*n* = 8/group); after a 30-min pretreatment with MPP (1 µM; n = *n* = 7–8/group), PHTPP (1 µM; *n* = 6–9/group), or G-15 (1 µM; *n* = 5–7/group); or with the MPP pretreatment (1 µM, 30 min) of cultures supplemented with E2 (1 µM) from DIV3 onward (*n* = 6–7/group). All data were analyzed as vehicle vs. oAβ by unpaired t-test (*P < 0.05, **P < 0.01)

To test this premise, we treated cultures (beginning at DIV3) with the aromatase inhibitor aminoglutethimide (AG) [53]. At 12–14 DIV, we assessed the impact of acute oAβ exposure (3–6 h/0.5 µM) on GIRK channel activity in HPC neurons with large soma and capacitance values (100–200 pF), as well as pyramidal morphology. In neonatal HPC cultures, these are characteristic features of CaMKIIα-positive excitatory/glutamatergic neurons [54–56], the HPC neuron subpopulation shown previously to exhibit the male-specific, oAβ-induced suppression of GIRK channel activity [19]. Using recording conditions that isolate and accentuate GIRK channel activity [19, 46, 57–59], we measured whole-cell currents evoked by ML297, a direct activator of GIRK1-containing GIRK channels [58], the dominant channel subtype in hippocampal neurons [60]. Subsequently, we measured G protein-dependent GIRK channel responses evoked by the GABA_B_ receptor-selective agonist baclofen. Strikingly, AG treatment unmasked the oAβ-induced suppression of GIRK channel activity in female HPC neurons (Fig. 1A-C).

Estrogen signaling is mediated by receptors including ERα and ERβ, known primarily as ligand-dependent transcription factors [61], as well as the G protein-coupled estrogen receptor GPER [62]. To identify the receptor(s) mediating the protective influence of estrogen in female HPC neurons, we next treated female HPC cultures with selective antagonists for ERα (methyl piperidino pyrazole/MPP [63, 64]), ERβ (PHTPP [65]), and GPER (G-15 [66]), for 30 min prior to exposure to oAβ or vehicle. While the ERβ antagonist PHTPP and GPER inhibitor G-15 were without effect, treatment of female HPC cultures with the ERα antagonist MPP unmasked the oAβ-induced suppression of GIRK channel activity in female HPC neurons (Fig. 1A-C). Furthermore, supplementation of culture medium with exogenous E2 beginning at DIV3 prevented the ability of MPP to unmask the oAβ-induced GIRK channel suppression in female HPC neurons (Fig. 1B, C). Collectively, these data indicate that E2/ERα prevents the oAβ-induced suppression of GIRK channel activity in female HPC neurons.

### Mechanism of the oAβ-induced suppression of GIRK channel activity in female HPC neurons

**Fig. 2:**
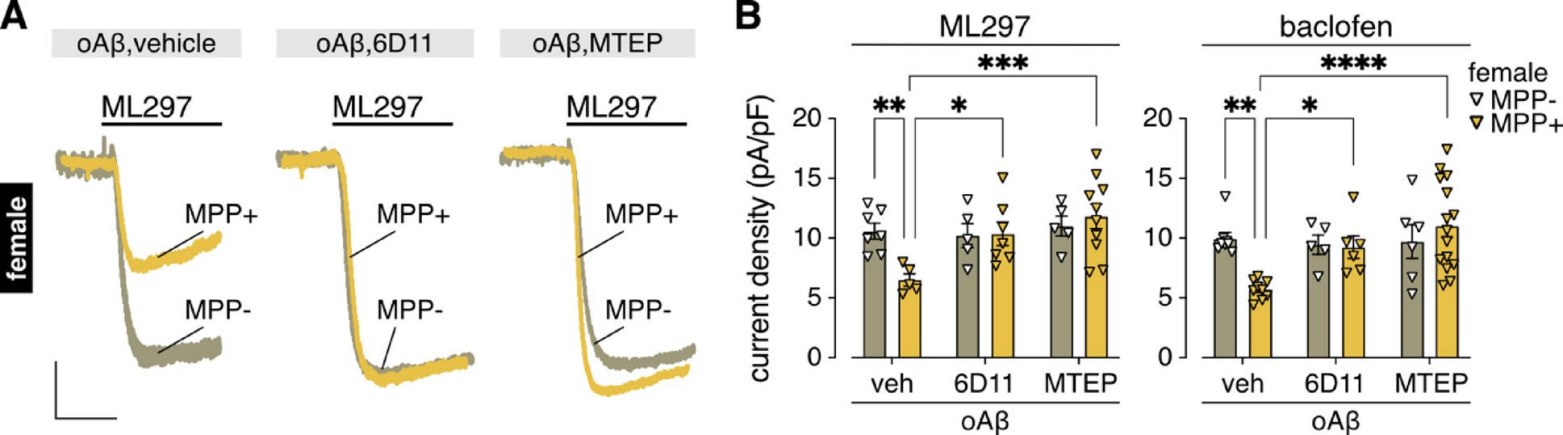
oAβ-induced suppression of GIRK channel activity in MPP-treated female HPC neurons involves PrP^C^/mGluR5 co-activation. **A**. representative currents evoked by ML297 (10 µM) in female HPC neurons treated with MPP (MPP+; 1 µM, 30 min) or vehicle (MPP-) prior to incubation with oAβ (0.5 µM, 3–6 h) and vehicle (left), the PrP^C^ antibody 6D11 (2.5 µg/mL, middle), or the mGluR5 antagonist MTEP (10 µM, right); scale: 500 pA/5 s. Only the first part of the traces showing the peak response is presented due to the long deactivation phase for ML297-induced currents. **B**. ML297 (10 µM, *n* = 5–10/group; left) and baclofen (100 µM, *n* = 5–9/group; right) current densities in female HPC neurons treated with (+) or without (-) MPP (1 µM, 30 min) prior to incubation with oAβ (0.5 µM, 3–6 h) and either vehicle (veh), 6D11 (2.5 µg/mL), or MTEP (10 µM). Data were analyzed by two-way ANOVA and Dunnett’s multiple comparisons (*P < 0.05, **P < 0.01, ***P < 0.001, ****P < 0.0001)

The oAβ-induced suppression of GIRK channel activity in male HPC neurons [19], like other forms of oAβ-induced neurotoxicity, requires co-activation of the cellular prion protein (PrP^C^) and metabotropic glutamate receptor 5 (mGluR5) [67]. As such, we next investigated whether the oAβ-induced suppression of GIRK channel activity unmasked in MPP-treated female HPC neurons also involves PrP^C^ and mGluR5. Consistent with our findings in male HPC neurons [19], oAβ-induced suppression of GIRK channel activity in MPP-treated female HPC neurons was blocked by the PrP^C^-specific antibody 6D11 and the mGluR5 receptor-selective antagonist MTEP (Fig. 2A, B). Importantly, neither 6D11 nor MTEP alone affected GIRK-dependent currents (SFig**. **1).

**Fig. 3:**
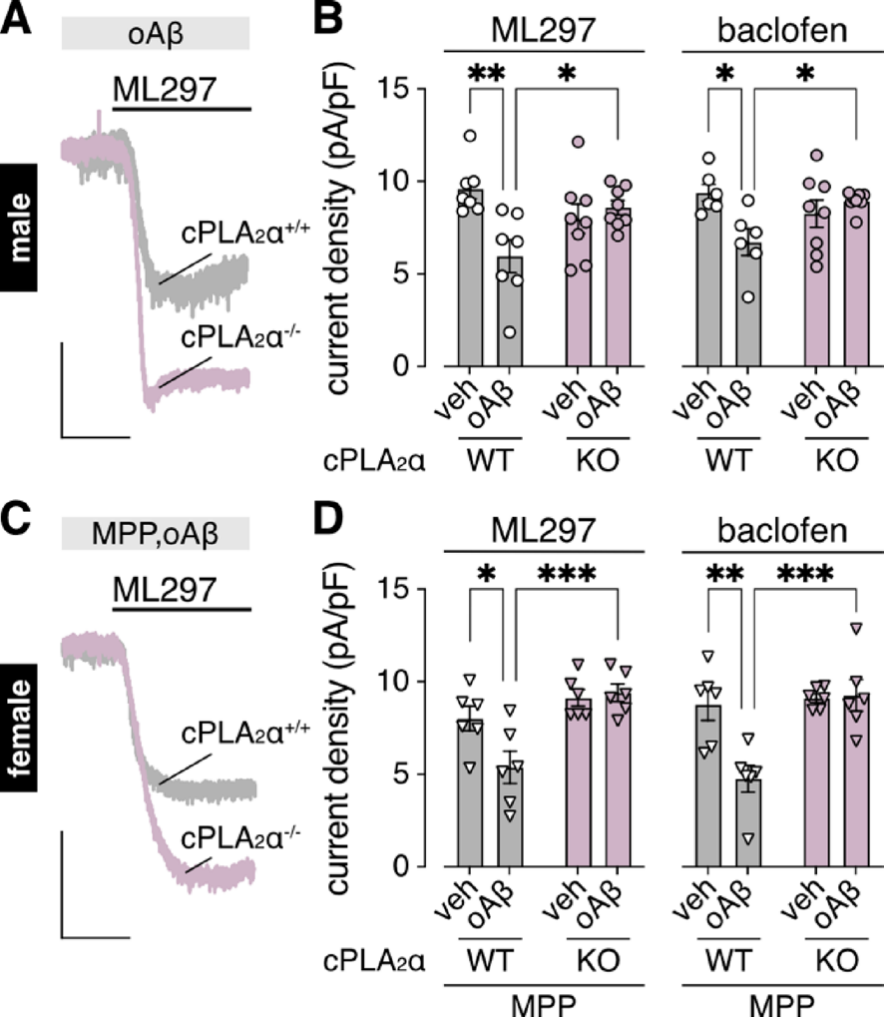
oAβ-induced suppression of GIRK channel activity in male and MPP-treated female HPC neurons requires cPLA_2_α.** A. **Representative currents evoked by ML297 (10 µM) in HPC neurons from male cPLA_2_α wild-type (+/+) and knockout (-/-) mice, neurons were treated with oAβ (0.5 µM, 3–6 h); scale: 500 pA/5 s. **B **.ML297 (10 µM; *n* = 7–8/group) and baclofen (100 µM; *n* = 6–8/group) current densities in HPC neurons from male cPLA_2_α wild-type (WT) or knockout (KO) mice, neurons were treated with either vehicle (veh) or oAβ (0.5 µM, 3–6 h); data were analyzed by two-way ANOVA and Šídák’s multiple comparisons (*P < 0.05, **P < 0.01). **C**. Representative currents evoked by ML297 (10 µM) in HPC neurons from female cPLA_2_α wild-type (+/+) and knockout (-/-) mice, neurons were treated with oAβ (0.5 µM, 3–6 h) following MPP pretreatment (1 µM, 30 min); scale: 500 pA/5 s. **D**. ML297 (10 µM) and baclofen (100 µM) current densities in HPC neurons (*n* = 6/group) from female cPLA_2_α wild-type (WT) or knockout (KO) mice, neurons were treated with either vehicle (veh) or oAβ (0.5 µM, 3–6 h) following MPP pretreatment (1 µM, 30 min); data were analyzed by two-way ANOVA and Šídák’s multiple comparisons (*P < 0.05, **P < 0.01, ***P < 0.001)

The oAβ-induced suppression of GIRK channel activity in male HPC neurons was blocked by pharmacological inhibition of cytosolic phospholipase A_2_α (cPLA_2_α) [19]. To build on these findings, we tested the impact of oAβ on HPC cultures prepared from constitutive cPLA_2_α knockout (cPLA_2_α^–/–^) mice [44]. The oAβ-induced suppression of GIRK channel activity was absent in HPC neurons from male cPLA_2_α^–/–^ mice (Fig. 3A, B), and in MPP-treated HPC cultures from female cPLA_2_α^–/–^ mice (Fig. 3C, D). Thus, in the absence of the protective influence of ERα, oAβ suppresses GIRK channel activity in female HPC neurons via the same PrP^C^-mGluR5-cPLA_2_α signaling pathway as in males.

### mGluR5 activation elicits a sex-dependent suppression of GIRK channel activity

**Fig. 4:**
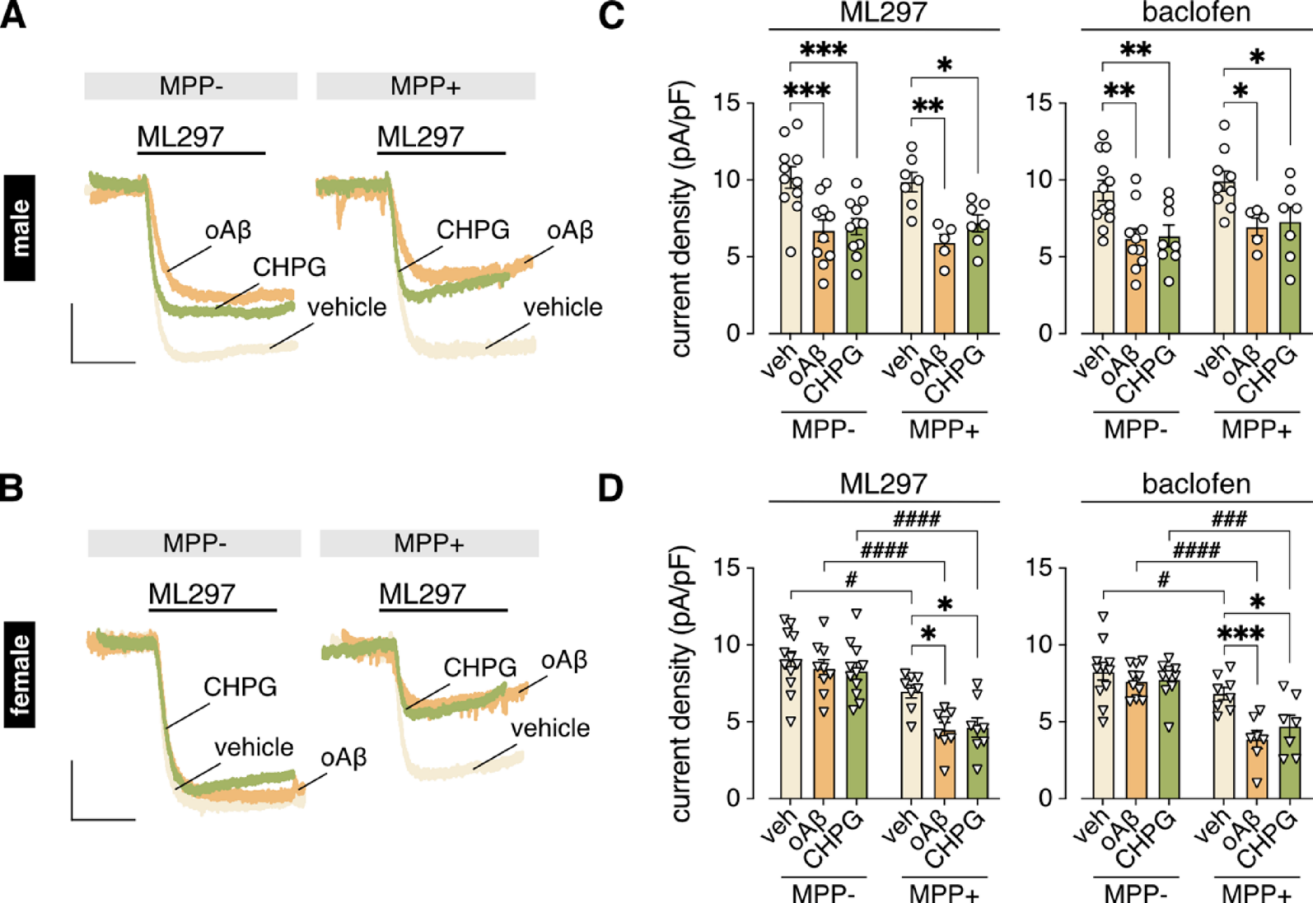
mGluR5 activation suppresses GIRK channel activity in male but not female HPC neurons. **A**. representative currents evoked by ML297 (10 µM) in HPC neurons from male mice 3–6 h after treatment with oAβ (0.5 µM), CHPG (1 mM), or vehicle, with or without MPP (1 µM, 30 min) pretreatment; scale: 500 pA/5 s. **B**. ML297 (10 µM; *n* = 5–11/group) and baclofen (100 µM; *n* = 5–12/group) current densities in male HPC neurons treated with vehicle (veh), oAβ (0.5 µM) or CHPG (1 mM), with or without MPP (1 µM, 30 min) pretreatment. Data were analyzed by two-way ANOVA and Tukey’s multiple comparisons within MPP treatment (*P < 0.05, **P < 0.01, ***P < 0.001). **C**. representative currents evoked by ML297 (10 µM) in HPC neurons from female mice 3–6 h after treatment with oAβ (0.5 µM), CHPG (1 mM), or vehicle, with or without MPP (1 µM, 30 min) pretreatment; scale: 500 pA/5 s. **D**. ML297 (10 µM; *n* = 9–12/group) and baclofen (100 µM; *n* = 7–12/group) current densities in female HPC neurons treated with vehicle (veh), oAβ (0.5 µM) or CHPG (1 mM), with or without MPP (1 µM, 30 min) pretreatment. Data were analyzed by two-way ANOVA and Tukey’s multiple comparisons within MPP treatment (*P < 0.05, ***P < 0.001) and within drug treatment (^#^*P* < 0.05, ^###^*P* < 0.001, ^####^*P* < 0.0001)

We next tested whether mGluR5 activation is sufficient to suppress GIRK channel activity in male and female HPC neurons. We treated HPC cultures with the mGluR5-selective agonist CHPG for 3–6 h, the same timeframe as oAβ treatment. CHPG treatment suppressed both ML297- and baclofen-evoked currents in male (Fig. 4A, B) but not female neurons (Fig. 4C, D), mimicking the sex-dependent effect of oAβ. Although MPP treatment did not impact the CHPG- or oAβ-induced suppression of GIRK channel activity in male HPC neurons (Fig. 4A, B), it did unmask the CHPG-induced suppression of GIRK channel activity in female HPC neurons (Fig. 4C, D). Thus, ERα in female HPC neurons protects GIRK channel activity from suppression by oAβ and mGluR5 activation. Interestingly, MPP treatment also decreased GIRK-dependent currents in vehicle-treated female neurons (Fig. 4C, D), suggesting that ERα exerts a positive tonic influence on GIRK channel activity in female HPC neurons (possibly by local estrogen production by aromatases).

**Fig. 5:**
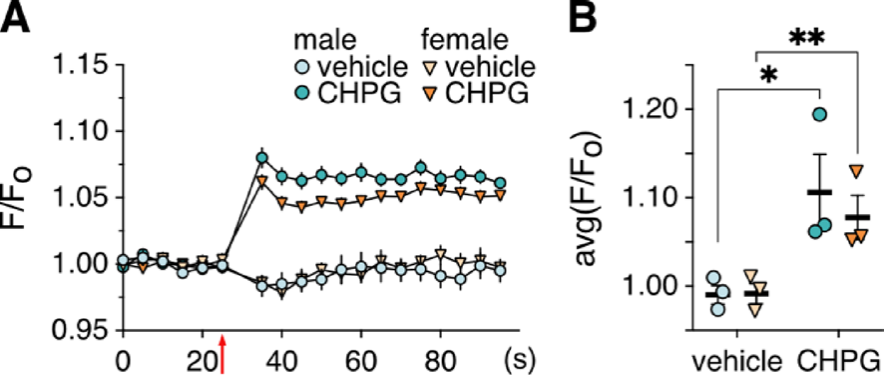
Impact of CHPG on intracellular Ca^2+^ in male and female HPC neurons. **A**. change in fluorescence (F/F_0_) associated with addition of vehicle or CHPG (1 mM) (denoted by the red arrow) to 1 male and 1 female HPC culture infected with AAVPHP.eB-CaMKIIα-jGCaMP8s. For this independent experiment, data at each timepoint represent the average F/F_0_ measured across 24 wells for each sex and treatment group. **B. **summary of the average change in fluorescence evoked by vehicle and CHPG (1 mM) in 3 independent experiments. In each independent experiment, an average F/F_0_ value for each well was calculated during the 60 s period after addition of vehicle or CHPG; this yielded 24 technical replicates per sex and treatment group. The data from 3 independent experiments were then analyzed by nested two-way ANOVA and Šídák’s multiple comparisons (*P < 0.05, **P < 0.01)

Activation of Group I mGluRs including mGluR5 stimulates G_q_, leading to PLC-mediated generation of inositol trisphosphate (IP_3_) and IP_3_ receptor-induced release of Ca^2+^ from the endoplasmic reticulum [68]. To test whether mGluR5-dependent intracellular Ca^2+^ mobilization also differs between male and female HPC excitatory/glutamatergic neurons, we used an AAV vector to drive expression of the cytosolic Ca^2+^ indicator jGCaMP8 under the control of the CaMKIIα-promoter. CHPG-induced increases in intracellular Ca^2+^ were comparable in male and female HPC cultures (Fig. 5), indicating that mGluR5 is expressed and functional in female HPC neurons and that it regulates intracellular Ca^2+^ dynamics to a similar degree as in male neurons. This finding also suggests that mGluR5-dependent regulation of intracellular Ca^2+^ is not required for either the CHPG- or oAβ-induced suppression of GIRK channel activity in male HPC neurons. Consistent with this premise, treatment of male HPC cultures with PLC inhibitors did not prevent the oAβ-induced GIRK channel suppression in male HPC neurons [19].

### Membrane-associated ERα confers resilience to mGluR5 activation in female HPC neurons

**Fig. 6:**
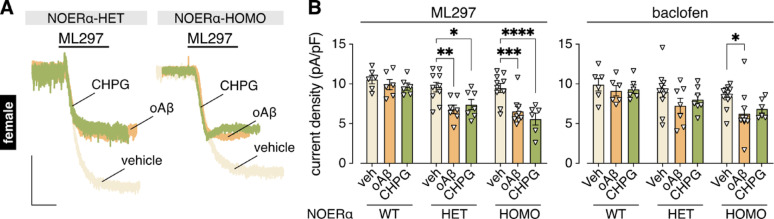
mERα confers resilience to the mGluR5-dependent suppression of GIRK channel activity in female HPC neurons. **A**. representative currents evoked by ML297 (10 µM) in female HPC neurons from heterozygous (HET) and homozygous (HOMO) NOERα knock-in mice following 3–6 h treatment with vehicle (veh), oAβ (0.5 µM), or CHPG (1 mM); scale: 500 pA/5 s. **B**. ML297 (10 µM; *n* = 6–10/group) and baclofen (100 µM; *n* = 6–11/group) current densities in HPC neurons from female NOERα wild-type (WT), heterozygous (HET), and homozygous (HOMO) knock-in mice following 3–6 h treatment with vehicle (veh), oAβ (0.5 µM) or CHPG (1 mM); data were analyzed by two-way ANOVA and Tukey’s multiple comparisons (*P < 0.05, **P < 0.01, ***P < 0.001, ****P < 0.0001, only the comparisons within treatment are presented)

A fraction of ERα in neurons is palmitoylated and trafficked to the plasma membrane, and membrane-associated ERα (mERα) can mediate rapid, non-genomic signaling [69, 70]. The non-genomic signaling via mERα in female HPC neurons is particularly intriguing given that mERα can mediate rapid E2 effects in rodent HPC cultures through association with and transactivation of Group I (mGluR1, mGluR5) and Group II (mGluR2, mGluR3) receptors [71, 72]. To determine if mERα confers resilience to the oAβ- and CHPG-induced suppression of GIRK channel activity in female HPC neurons, we generated cultures from nuclear-only ERα knock-in (NOERα-KI) mice. To determine if mERα confers resilience to the oAβ- and CHPG-induced suppression of GIRK channel activity in female HPC neurons, we generated cultures from nuclear-only ERα knock-in (NOERα-KI) mice [43]. This mouse line expresses normal amounts of nuclear ERα and exhibits normal genomic ERα signaling; however, these mice have a 1-amino acid mutation activity in ERα that precludes the post-translational palmitoylation that causes ERα to localize to the cell membrane, thus have no expression of mERα or mERα signaling despite normal genomic ERα-mediated E2 signaling [43]. This model has been extensively used to determine relative roles of nuclear versus membrane ERα. In HPC neurons from heterozygous and homozygous NOERα-KI female mice, oAβ and CHPG both suppressed ML297- and baclofen-induced GIRK currents (Fig. 6A, B).

**Fig. 7:**
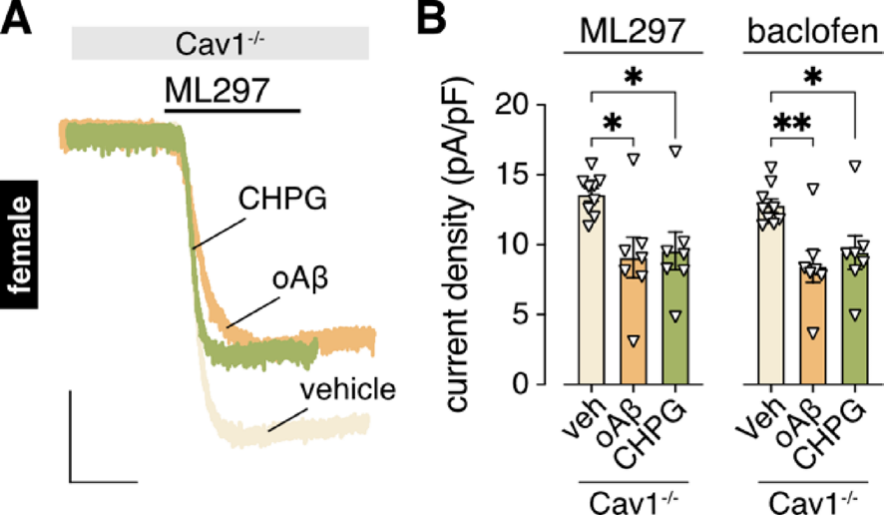
Cav1 confers resilience to the mGluR5-dependent GIRK channel suppression in female HPC neurons. **A**. representative currents evoked by ML297 (10 µM) in HPC neurons from female Cav1 knockout (-/-) mice following 3–6 h treatment with vehicle (veh), oAβ (0.5 µM), and CHPG (1 mM), respectively; scale: 500 pA/5 s. **B**. ML297 (10 µM) and baclofen (100 µM) current densities in HPC neurons (*n* = 7–9/group) from female Cav1 knockout (-/-) mice following 3–6 h treatment with vehicle (veh), oAβ (0.5 µM) or CHPG (1 mM); data were analyzed by one-way ANOVA and Tukey’s multiple comparisons (*P < 0.05, **P < 0.01)

In the rodent HPC, complex formation between mERα and Group 1 mGluRs (mGluR1 and mGluR5) requires the scaffolding protein caveolin-1 (Cav1) [72]. If association with mGluR5 underlies the resilience conferred by mERα, therefore, loss of Cav1 should unmask the oAβ-induced suppression of GIRK channel activity in female HPC neurons. Indeed, both oAβ and CHPG suppressed ML297- and baclofen-induced GIRK currents in MPP-treated female HPC cultures from Cav1^–/–^ mice (Fig. 7A, B). Collectively, these data suggest that mERα/Cav1 confers resilience to the oAβ-induced and mGluR5-dependent suppression of GIRK channel activity female HPC neurons.

## Discussion

**Fig. 8:**
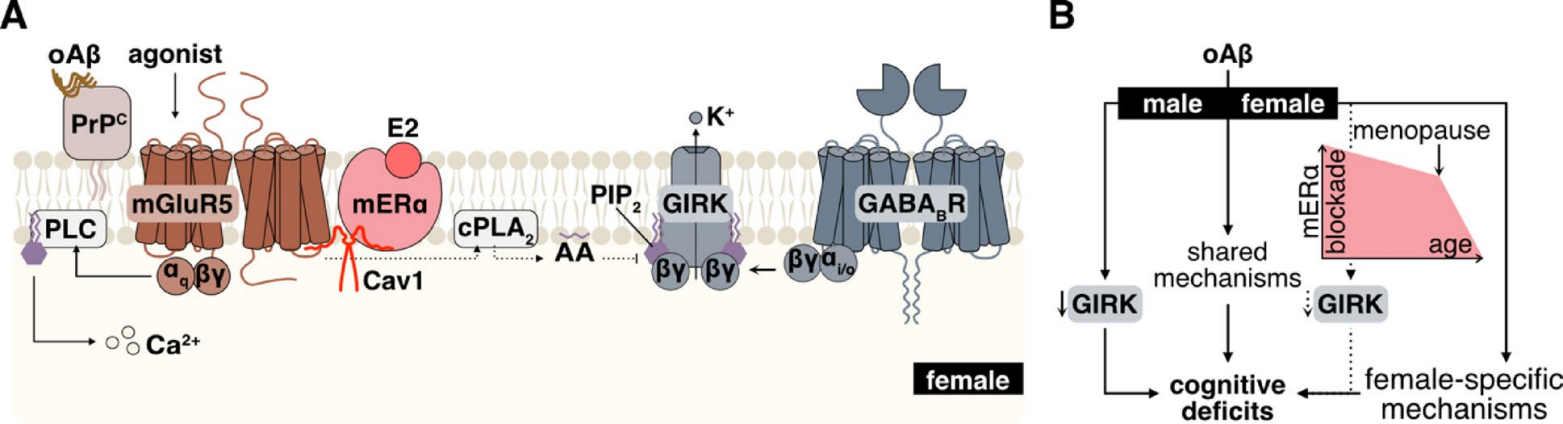
Model for the protective influence of mERα/Cav1 on GIRK channel activity in female HPC neurons. **A**. In male HPC neurons, oAβ inhibits GIRK channel activity and GIRK-dependent inhibitory GPCR signaling by activating a PrP^C^–mGluR5–cPLA_2_ pathway, which increases AA levels and disrupts the PIP_2_-GIRK channel interaction. In female HPC neurons, although mGluR5-mediated PLC-IP_3_-Ca^2+^ signaling is comparable to that in males, the oAβ-induced suppression of GIRK channel activity is blocked by mERα, in the presence of E2 and Cav1. When this resilience factor is removed, oAβ suppresses GIRK channel activity in female HPC neurons via the same PrP^C^-mGluR5-cPLA_2_ pathway as in male neurons. **B**. oAβ-induced suppression of neuronal GIRK channel activity contributes to synaptic and behavioral deficits linked to cognitive dysfunction in male AD models. Although mERα/Cav1 blocks the GIRK channel suppression in females, oAβ can still induce cognitive deficits through GIRK-independent mechanisms. As E2 levels decline with aging and especially menopause, reduced mERα/Cav1-mediated resilience may allow oAβ to suppress GIRK channel activity in female neurons as well, potentially accelerating disease progression

oAβ suppresses GIRK channel activity in HPC neurons from male mice via a PrP^C^-mGluR5-cPLA_2_α signaling pathway [19]. Here, we show that mERα/Cav1 confers resilience to this oAβ-induced neuroadaptation in female HPC neurons. When the protective influence of mERα/Cav1 is blocked or absent, oAβ suppresses GIRK channel activity in female HPC neurons via the same PrP^C^-mGluR5-cPLA_2_α signaling pathway identified in male neurons (Fig. 8A).

Clinical observations related to early mild cognitive impairment in Alzheimer’s disease show that women initially outperform men in cognitive tasks [73–75], but then exhibit faster decline [76, 77]. This suggests a “tipping point” phenomenon wherein women better preserve function early in AD, but decline accelerates once a pathological threshold is surpassed [75]. Notably, menopause onset coincides with the early stages of AD pathogenesis in women [78], and earlier menopause is linked to increased cognitive decline [79, 80] and AD susceptibility [81, 82]. Premature surgical menopause, such as bilateral oophorectomy, can increase AD risk by up to 70% [83]. As neuroendocrine transition associated with menopause can hasten or exacerbate early disease processes, perimenopause represents a critical window for AD prevention [84]. Indeed, estrogen replacement therapy (ERT) during this window appears especially neuroprotective [85, 86].

Our findings are consistent with a protective role of E2. Inhibition of endogenous E2 synthesis via aromatase blockade rendered female HPC neurons susceptible to oAβ-mediated GIRK channel suppression, whereas E2 supplementation restored resilience, likely by overcoming MPP antagonism of mERα. Our study underscores the importance of considering sex as a biological variable in neonatal culture models. The efficacy of the aromatase inhibitor AG in the HPC culture model suggests that local estrogen production can explain a striking functional sexual dimorphism, in this case the resilience of GIRK channel activity in female HPC neurons to oAβ.

Previously, we reported that infusion of oAβ into the dorsal HPC evoked cognitive deficits in both male and female mice, but this manipulation only suppressed GIRK-dependent signaling in male CA1 pyramidal neurons [19]. These findings were paralleled in the well-studied APP/PS1 transgenic mouse AD model; aged (12 month) mice of both sexes exhibited cognitive deficits, but only male subjects exhibited diminished GIRK channel activity in CA1 pyramidal neurons [19]. Thus, the suppression of GIRK channel activity in HPC neurons appears to contribute to early cognitive impairment in male mouse AD models, while non-GIRK mechanisms drive impairment in female models. While aging female mice eventually transition to an anestrous state, reproductive senescence for most involves a transitional state characterized by sustained levels of E2 that can last for months [87]. We speculate that mERα-mediated resilience diminishes as E2 levels decline with aging and menopause. The combined effect of female-specific pathological mechanisms and the unmasked oAβ-induced suppression of GIRK channel activity then accelerates and exacerbates disease progression (Fig. 8B). Since loss of GIRK channel activity in females emerges at later stages, when compensatory mechanisms are compromised, the impact of this neuroadaptation on cognitive decline could even be more profound.

mERα mediates rapid effects of exogenous E2 in rodent HPC cultures through transactivation of Group I (mGluR1, mGluR5) and Group II (mGluR2, mGluR3) receptors [71]. mERα interactions with Group I and Group II mGluRs require caveolin-1 (Cav1) and caveolin-3 (Cav3), respectively [72]. Cav1 has been implicated in AD [88, 89]. For example, young (3–6-month) Cav1^–/–^ mice show elevated Ab and tau deposition in the HPC, as well as several signs of premature neuronal aging and degeneration [90]. In addition, gene delivery of Cav1 in the HPC preserved dendritic arborization, synaptic ultrastructure, axonal myelination, and cognitive performance in a transgenic AD mouse model [91]. The unmasking of the oAβ- and CHPG-induced suppression of GIRK channel activity in female HPC neurons from NOERα-KI and Cav1^–/–^ mice indicates that mERα/Cav1 is critical in conferring resilience in female neurons.

E2 induced an mERα/Cav1-dependent transactivation of mGluR1 in female but not male rat HPC cultures, leading to activation of the transcription factor cAMP response element-binding protein (CREB) [71, 72]. Despite this finding, we consider mERα/Cav1-dependent transactivation of mGluR1 to be an unlikely mechanism underlying the resilience of GIRK channel activity of oAβ. Indeed, the mGluR1-selective antagonist LY36785 did not unmask the oAβ-induced suppression of GIRK channel activity in female HPC cultures (SFig. 2). Although E2-associated CREB activation was shown to be mGluR5-independent in the HPC [71], the E2-induced, mERα transactivation of mGluR5 was reported in striatal neurons [92]. Thus, mERα/Cav1-dependent transactivation of mGluR5 could occlude the oAβ-induced suppression of GIRK channel activity in female HPC neurons. In this case, the mGluR5 antagonist MTEP should enhance baseline GIRK channel activity in female but not male neurons. However, MTEP had no effect on GIRK channel activity in female HPC neurons (SFig. 1), and GIRK-dependent currents measured in MPP-treated female neurons were smaller than those in untreated controls (Fig. 4C, D). Thus, we consider mERα/Cav1-dependent transactivation of mGluR5 to be an unlikely explanation for resilience in female neurons as well.

Resilience of GIRK channel activity to oAβ in female HPC neurons could involve an mERα/Cav1-dependent disruption of the PrP^C^-mGluR5 interaction. Consistent with this premise, mGluR5 co-immunoprecipitated with PrP^C^ in HPC tissue from male but not female mice [93]. oAβ enhances PrP^C^-mGluR5 co-immunoprecipitation, but the enhancement requires intact lipid rafts [94]. As a key adaptor protein associated with lipid rafts [95], Cav1 appears to serve as a critical interaction hub among these signalosome elements. In addition to ERα [96], Cav1 can bind to PrP^C^ [97], Aβ_1–42_ [98], and to the intracellular domain of Group I mGluRs [99]. Interestingly, a critical structural determinant (S522) of the ERα-Cav1 interaction [72, 96] is adjacent to the orthosteric binding site within ERα [100]. Thus, MPP could disrupt the interaction between Cav1 and mERα, weakening the association of the mERα/Cav1 complex with mGluR5; this might allow for increased interaction between mGluR5 and PrP^C^ (Fig. 8A). Notably, in the lipid raft fractions from human cortices, mERα, Cav1, PrP^C^, and mGluR5 have all been identified as components of the “ER-related signalosome”, a structural organization disrupted in menopause and late-stage AD [101, 102].

Given that oAβ (and CHPG) suppress GIRK channel activity to a comparable extent in neurons from heterozygous and homozygous female NOERα-KI mice, resilience to oAβ and CHPG in female HPC neurons may be linked to a relatively modest sex difference in effective mERα levels. In this regard, it is notable that while E2 is not necessary for the membrane localization of mERα, it can promote ERα trafficking to the membrane [103] and enhance association with mGluRs [104]. In addition, a co-immunoprecipitation study revealed 2-fold higher level of mERα/mGluR5 complexes in female compared to male mouse cortex [105], and extranuclear ERα is more abundant in the female HPC [106].

Pharmacological inhibition of cPLA_2_α prevented the oAβ-induced suppression of GIRK channel activity in male HPC neurons [19]. These findings are consistent with published studies showing that the non-selective Group 1 mGluR agonist DHPG suppressed GIRK-dependent signaling in HPC pyramidal neurons via PLA_2_-mediated generation of arachidonic acid (AA) [107, 108], which can disrupt the interaction between the GIRK channel and PIP_2_ [109]. Indeed, intracellular perfusion of the PIP_2_ analog diC8PIP_2_ rescued the oAβ-induced suppression of GIRK channel activity in male HPC neurons [19]. cPLA_2_α, also known as group IVA PLA_2_ (GIVA-PLA_2_) [110], has been implicated in other forms of Aβ-induced neurotoxicity [111–113]. For example, pharmacological inhibition of cPLA_2_α reduced Aβ-induced neurotoxicity in HPC cultures and ablation of cPLA_2_α improved Morris Water Maze performance in the J20 mouse AD model [112]. We show here that ablation of cPLA_2_α prevents the oAβ-induced suppression of GIRK channel activity in male HPC neurons, and in female HPC neurons treated with MPP.

PrP^C^/mGluR5 co-activation has been implicated in the oAβ-induced increase in intracellular Ca^2+^ in neurons [114], and cPLA_2_α can be activated by increased intracellular Ca^2+^ [115]. We show here that mGluR5-selective agonist CHPG evokes a comparable increase in intracellular Ca^2+^ in male and female HPC neurons but only suppresses GIRK channel activity in male neurons. This argues that increased intracellular Ca^2+^ is not involved in the oAβ-induced activation of cPLA_2_α in cultured HPC neurons. cPLA_2_α can also be activated by phosphorylation at multiple residues and via multiple kinases [110]. A potential role for p38 mitogen-activated protein kinase (p38 MAPK) in the stimulation of cPLA_2_α is particularly intriguing given evidence that p38 MAPK activation via an mGluR5-dependent mechanism has been implicated in oAβ-induced neurotoxicity in the rat HPC [116].

We would like to highlight important limitations of the current study. The main goal was to understand a striking sex difference in the susceptibility of male and female cultured HPC neurons to the oAb-induced suppression of GIRK channel activity [19]. Our findings highlight an intriguing form of resilience in female HPC neurons that is conferred by ERa and Cav1, factors that have garnered attention in the context of AD [101, 102]. The impact of acute administration of exogenous oAb on signaling pathways in neonatal cultured neurons, however, does not effectively model the gradual accumulation of pathological factors, including oAb and hyperphosphorylated tau proteins, that drives the age-related neurophysiological impairments in AD. Moreover, to measure the impact of oAb on GIRK channel activity in HPC neurons, we used recording conditions designed to isolate and accentuate non-physiological inward K^+^ currents through these channels. While we have shown that acute exogenous oAb suppresses the physiologically relevant GIRK-dependent outward currents in CA1 pyramidal neurons in HPC slices to a similar degree as in HPC cultures [19], the extent to which this adaptation impacts the excitability of these and other neurons in the AD brain is unclear. Given that manipulation of GIRK channel activity (enhancement or suppression) in CA1 pyramidal neurons disrupts multiple aspects of cognitive function in mice [21], we speculate that even modest disruptions of GIRK channel activity could contribute to cognitive impairments observed in AD.

## Conculusions

Sexually divergent phenotypes related to glutamatergic neurotransmission, and mGluR5-dependent signaling specifically, are increasingly recognized [117–122]. Our findings add to this growing body of evidence by elaborating a male-specific, mGluR5-dependent neuroadaptation in inhibitory signaling relevant in mouse AD models and identifying female-specific factors that confer resilience to this neuroadaptation. The insights gained may guide the development of sex-specific and mechanism-informed treatment strategies for AD.

## Supplementary Information

Supplementary Material 1.

## Data Availability

All data needed to evaluate the conclusions in the paper are present in the paper or the Supplementary Materials. The datasets used and/or analyzed, as well as viral vector sequences and protocols used as part of the current study, are available from the corresponding author on reasonable request.
