# Supplementary material for: Membrane-associated estrogen receptor α prevents the amyloid β-induced suppression of GIRK channel activity in hippocampal neurons from female mice
Source: Biol Sex Differ. 2025 Nov 6;16:90. doi: 10.1186/s13293-025-00776-7 (PMC12593801; doi:10.1186/s13293-025-00776-7)
Supplement: Supplementary file 1 — Supplementary Material 1. [file 13293_2025_776_MOESM1_ESM.docx]

**Membrane-associated estrogen receptor α prevents the amyloid β-induced suppression of GIRK channel activity in hippocampal neurons from female mice**

Haichang Luo^a^, Ezequiel Marron Fernandez de Velasco^a^, Jaeyoon Kim^a^, Praseuth Yang^a^, Paul Mermelstein^b^, Joseph V. Bonventre^c,d^, Paul S. Cooke^g^, Kevin Wickman^a*^

**SUPPLEMENTAL MATERIAL**

**SUPPLEMENTAL FIGURE 1**

Impact of 6D11 and MTEP on baseline GIRK channel activity in female HPC neurons

**SUPPLEMENTAL FIGURE 2**

mGluR1 inhibition does not reveal the oAβ-induced suppression of GIRK channel activity in female HPC neurons

**Supplemental Figure 1. Impact of 6D11 and MTEP on baseline GIRK channel activity in female HPC neurons.** Summary of ML297 (10 µM) and baclofen (100 µM) current densities in female HPC neurons (n=5-9/group) treated with vehicle (veh), 6D11 (2.5 µg/mL), or MTEP (10 µM) for 3-6 h. Data were analyzed by one-way ANOVA (ns: P>0.05).

**Supplemental Figure 2. mGluR1 inhibition does not reveal the oAβ-induced suppression of GIRK channel activity in female HPC neurons.** Summary of ML297 (10 µM, n=6-9/group) and baclofen (100 µM, n=5-9/group) current densities in female HPC neurons treated with vehicle (veh) or oAβ (0.5 µM, 3-6 h), measured following 30-min pretreatment with a selective mGluR1 antagonist LY367385 hydrochloride (10 µM).
